# Fall risks and the related factors for the homebound older people with dementia: Evidence from East China

**DOI:** 10.3389/fpubh.2022.946097

**Published:** 2022-08-25

**Authors:** Xiaoxin Dong, Guanjun Liu, Xiaoxu Yin, Rui Min, Yueming Hu

**Affiliations:** ^1^Institute of Health Services, Ningbo College of Health Sciences, Ningbo, China; ^2^School of Public Health, Tongji Medical College, Huazhong University of Science and Technology, Wuhan, China; ^3^Department of Orthopedics, Hwa Mei Hospital, University of Chinese Academy of Sciences, Ningbo, China; ^4^Ningbo Institute of Life and Health Industry, University of Chinese Academy of Sciences, Ningbo, China

**Keywords:** fall risk, older people, dementia, homebound care, community, morse fall risk scale

## Abstract

**Purpose:**

Falls are a major public health problem, especially for older people. This research aimed to provide a direct illustration of fall risks among the homebound older people with dementia in China, and to identify the risk factors associated with it.

**Methods:**

In 2020, a questionnaire-based field survey was used to assess 1,042 people aged over 60 years in Ningbo, Eastern China. The Morse Fall Risk Scale's result was employed as the dependent variable, while the basic health problems, living environment difficulties, social support problems, and behavioral awareness issues were utilized as the independent variables; subsequently, chi-squared tests and four multivariate ordinarily ordered logistic regression models were performed.

**Results:**

Overall, nine hundred and thirty-one older people with dementia were included in this study (the effective rate was 89.34%), with the majority of them having severe dementia (27.9%). Furthermore, 16.2% had fallen in the past 3 months, and 16.8% were at a high risk of falling. The risk factors for the older people's cognitive function included 80–90 years old, vascular dementia, marital status, and history of falls (*P* < 0.05); the kinds of chronic diseases, the activities of daily living, living environment, caregiver burden, caregiver knowledge, the Cohen Mansfield Agitation Inventory results, and the Clinical Dementia Rating were the protective factors for the risk of falls in them (*P* < 0.05).

**Conclusion:**

The risk of falling of the Chinese homebound older people with dementia was high. Their caregivers, such as relatives, need to pay attention to these risk factors and perform appropriate measures to prevent falls.

## Introduction

There are over 55 million people living with dementia worldwide; further, this number is predicted to reach 78 million in 2030 (1). Dementia not only manifests as the weakening of physical function, but also the gradual loss of neurological functions, such as memory and orientation, accompanied several mental and behavioral symptoms (2). Notably, home care remains the dominant care mode internationally for the older people with dementia (OPWD). According to the statistics released by the Alzheimer's Disease International, over 70% of the OPWD live at home, and also wish to remain there (3). Specifically, in China, more than 80% of them are cared for at home (4).

However, the homebound OPWD are subject to several safety issues. For example, it has been reported that 30–50% of them have experienced falls (5, 6). Herein, a high rate of falling is related to a poor self-care ability and the overall orientation that are common among the OPWD (6); additionally, an unreasonable room layout renders their living environment unsafe (7). According to the World Health Organization (WHO), a fall is defined as an event that results in a person coming to rest inadvertently on the ground, the floor, or other lower levels. Globally, falls are a major public health problem. An estimated 684,000 fatal falls occur yearly, making it the second leading cause of an unintentional injury death, with the greatest number occurring in the adults aged above 60 years (8). According to the data analysis of China's disease surveillance system, falls have become the first direct cause of death in the Chinese people aged over 65 years (9). In addition, those individuals who fall and suffer a disability, particularly the elderly, are at a major risk of requiring subsequent long-term care and institutionalization. The OPWD often experience contusion bleeding, joint dislocation, or sprained ligaments after falling; additionally, in severe cases, the lower limbs and the hip may be fractured. If a fracture occurs, due to long-term bed rest, it would also lead to the local skin compression, pressure sores, and even risks such as deep vein thrombosis, hypoproteinemia, and sepsis of infection that seriously threaten the older people's health (10).

In the 21st century, population aging has become a chief problem experienced globally; it entails a corresponding surge in the proportion of the older people in the total population due to a decrease and/or an increase in the number of the younger and older individuals, respectively. According to the data of the seventh national census in 2020, the number of older people aged 65 years and above in China has reached 190.64 million, accounting for 13.5% of the total population. Furthermore, 38.9% of the elderly aged 65 years and over have an unsound cognition (11). China is experiencing an accelerated period of population aging (12). It has increased the number of older people needing life care, thus growing the demand for the medical and health services as well as the burden of family pensions and the social old-age and medical security. Regarding the trend of population aging, such as the increase in the prevalence of chronic diseases in the elderly, problems such as life self-care disorders and a loss of cognitive function have become common social phenomena; moreover, a considerable number of older people still have different degrees of depression and a reduced social support (13).

To cope with the population aging, countries have made several attempts worldwide. The United Kingdom (UK) is one of the earliest countries globally to enter an aging society; the education for older people began earlier and developed relatively well, with rich educational resources and several informative channels, including the higher education, local education, and volunteer group organization systems. The elderly can participate in education in the higher and local government education systems together with the individuals of other ages as ordinary adults, as well as in the education specifically organized for them by voluntary groups. The UK's third-age universities have an extremely significant impact on the older persons' global education (14). China's older people industry started late compared with the foreign countries; moreover, most of it has been directly managed by the government agencies. Currently, home care provided by spouse and offspring is the most important source of care for the older people with functional disabilities in China. Due to various objective reasons, the proportion of home care for the Alzheimer's patients in China is high. They are prone to accidents such as falls, aspiration, burns, and losses; further, the care burden is substantial.

In the United States, falls are the foremost cause of accidental deaths and the 7th leading reason for deaths among people aged more than 65 years. Currently, some studies have been conducted to prevent falls in nursing homes, however, few have focused on the safety risks of the OPWD receiving home care. In reality, 30–40% and 50% of the seniors living in the community and the nursing homes, respectively, fall yearly (15). The damage of the older people falling at home is equally great. Assessing the risk factors associated with falls in the patients with dementia can prevent them and also reduce the burden. Therefore, this study analyzed the homebound OPWD, evaluated the risk factors of their falls, and aimed to improve their health and safety.

## Methods

### Study design

In 2020, a randomly selected field survey was carried out in three communities in the Ningbo City, Zhejiang Province, Eastern China to investigate the risk of home-based falls regarding the OPWD. It was conducted by the medical students and the staff who worked in the community health service centers after receiving a systematic training. The research was certified by the local ethics committee.

According to the truncated statistics, the elderly's population in the province has continued to grow in recent years. From the end of 2010 to that of 2020, the total number of older people increased from 7.8903 to 11.8752 million, a net increase of 3.9849 million in 10 years, with an average annual growth rate of 4.17%; further, the proportion of this population increased from 16.6 to 23.43%. The total number of older people aged 80 years and above grew from 1.2109 to 1.7765 million, a net increase of 565,600 in 10 years, with an average annual growth of 3.91%. The Zhejiang Province pays great attention to the elderly's quality of life; moreover, 2,674 medical institutions in the province provide door-to-door medical and health services for such individuals at home, and 9 units practices have been selected as the “National Typical Experience in the Integration of Medical and Nursing Care.” As of the end of 2020, 66,800 online nursing specialist clinics and 7,348 home nursing services have been conducted, thus solving the urgent needs of numerous patients. Simultaneously, 683 township home care service centers have been built (16).

### Inclusion criteria

The inclusion criteria for the respondents were as follows: a. Local Census over 60 years old; b. Conforms with the Diagnostic and Statistical Manual for Mental Disorders Fifth Edition diagnostic criteria for dementia (DSM-V) and is diagnosed by psychiatrists; c. Mainly receiving care at home; d. The main caregivers are family members; e. The caregivers understand the older people's condition and daily behavior; f. The caregivers can communicate normally; g. Willing to participate in the survey.

### Sampling

We conducted a survey on the needs of the homebound older people in the Ningbo City, from which the relevant persons with a clear diagnosis or self-complaint of dementia were screened. The sample size was calculated according to the survey sample size estimation formula of the current survey rate, which was as follows:


$$\begin{array}{l} n=\frac{Z_{\alpha /2}^{2}P\left(1-P\right)}{\delta^{2}} \end{array}$$

Here, *P* represents the sample rate; *Z*_α_ is the Z boundary value of the normal variable corresponding to the bilateral tail area under the standard normal distribution. The significance level was set at 0.05, and thus, *Z*_α/2_ = 1.96. The allowable error δ was set to 0.15 P.

According to the existing literature, the fall incidence in the home care for the OPWD in China in recent years is 30–50%. In order to ensure the sample's representativeness as far as possible, this study calculated the sample size according to the risk incidence of 30% (P), and concluded it to be 398 OPWD and caregivers. Considering the existence of the invalid questionnaires, the total number of samples required was at least 443 OPWD at a 10% missing rate. Finally, there were 1,042 questionnaires in total, excluding more than 10% of those with missing information (98 questionnaires) and with obvious inconsistencies (13 questionnaires); subsequently, 931 valid questionnaires remained. The effective rate was 89.34%.

### Variables

This study was conducted using a self-made questionnaire based on the maturity scales. The questionnaire included four parts: the basic situation of the older people, the assessment of risk factors, the evaluation of social support, and the basic information of the main caregiver. We introduced some internationally accepted scales to assess the risk factors, such as the activities of daily living (ADL), the Clinical Dementia Rating (CDR), the Morse Fall Risk Scale (MFS), the Cohen Mansfield Agitation Inventory (CMAI), the Zarit Burden Interview (ZBI), and more. We divided these variables into four aspects. The overall questionnaire had a Cronbach's α value of 0.907.

The MFS consists of six entries; the entries and the scoring criteria are as follows: (1) History of falling; immediate or within 3 months (no = 0 points, yes = 25 points); (2) Secondary diagnosis (no = 0 points, yes = 15 points); (3) Ambulatory aid (Bed rest/nurse assist = 0 points, Crutches/cane/walker = 15 points, Furniture = 30 points); (4) IV/Heparin Lock (no = 0 points, yes = 20 points); (5) Gait/Transferring (Normal/bedrest/immobile = 0 points, Weak = 10 points, Impaired = 20 points); (6) Mental status (Oriented to own ability = 0 points, Forgets limitations = 15 points). With an overall score of 125, the higher the score, the greater risk of falls (17). The Chinese version of the MFS was feasible; the Cronbach's α was 0.086 (18).

#### Basic health issues

The basic situation of the patients included gender, age, whether they have been diagnosed with dementia, types of dementia, anti-dementia drugs, and the number of chronic diseases. ADL has 10 items (Feeding, taking a bath, decorating, dressing, control stool, control urination, toileting, bed and chair transfer, walking on the ground for 45 meters, going up and down stairs). According to the independent completion's degree of the respondents, it could be divided into no complications; difficulties, however, can still be completed, problems that need help; and those that cannot be completed, counted as one to four points, respectively. According to the Barthel index score, the ability to perform the ADL was divided into three levels: good, medium, and poor. More than 60 points represent good ability; specifically, there is a mild dysfunction, and the individual can independently complete part of the daily activities and needs partial assistance. Further, 41 to 60 points denote moderate dysfunction; in particular, the people need considerable help to complete the ADL. Lastly, equal to or < 40 points indicate poor ability; there is severe dysfunction, and most ADL cannot be completed or require the service of others (19).

#### Living environment issues

The living environment questionnaire consists of eight entries, eight entries, each of which is rated on a five-point Likert scale ranging from completely incapable to completely able. This is used to assess whether the home environment can provide the basic living conditions for the OPWD and reduce the safety risks (20).

#### Social support assessment

The social support assessment comprised the marital status, the education level, the average monthly income, whether they live alone, the type of medical insurance, the number of children, the education level of the caregiver, and the caregiver's knowledge of dementia.

The ZBI has 4 dimensions, including caregiver health, mental state, and economic and social life. There are a total of 22 items, each of which is scored from 0 to 4 points; a score of 21 to 40 and 41 to 60 points indicate no or mild burden and moderate or heavy burden, respectively (21).

#### Behavioral awareness issues

The CMAI has 3 factors and 36 entries. According to the number of symptoms, it is divided into 7 grades (no, less than once weekly, once or twice weekly, several times weekly, once or twice daily, several times daily, and many times hourly = 1, 2, 3, 4, 5, 6, and 7 points, respectively) (22).

The CDR includes skills regarding memory, orientation, judgment and problem-solving, work and social interaction abilities, family life and personal hobbies, and independent life capabilities. All aspects of these six functions are assessed from no to serious injury; although the scores of each function are not superimposed, their assessment is synthesized into a total score according to the overall scoring criteria. The results are expressed as 0, 0.5, 1, 2, and 3 points, judging as normal, suspicious, mild, moderate, and severe dementia, respectively (23). In addition, we assessed if there was a history of falling, aspiration by mistake (24), scalding, and falling from the bed in the past 3 months.

### Quality control

A relevant training was conducted before and during the project, with a cumulative duration of 3 h. In the training, professionals were invited to explain the problems involved in the questionnaire and the content as well as the scale's precautions and survey skills; additionally, the investigators who participated in the training were invited to demonstrate. A face-to-face approach was employed to explain this survey's purpose and precautions to the older people and their families. The survey emphasized the principle of confidentiality and was conducted in the form of oral questions; the investigators filled out the questionnaires and two people in each group conducted the survey. Attention was paid to checking when the questionnaire was recycled. The questionnaires were entered by the investigators using the SPSS software. Before the statistical analysis, the data was organized and the invalid questionnaires were eliminated; after the data entry, the double-check method was adopted to verify the data for a second time to ensure the data entry's accuracy.

### Statistical methods

After the data collection was completed, the SPSS database was established; the SPSS 25.0 software was used for the statistical description and data analysis. The basic descriptive statistical values for all variables were provided. The *t*-test/chi-squared test was employed to determine whether each variable affected the fall risk score of the older people in China (*P* < 0.05). Subsequently, taking the relevant factors screened out as the independent variables and the fall risk score scale (“low,” “medium,” and “high risk of falls”) as the dependent variable, the variables were successively grouped into four models. The four multivariate ordinarily ordered logistic regression models were established to assess the correlation with the falls. The odds ratio (OR) was calculated from the model results. The test level was α = 0.05.

## Results

### Basic information about the risk of falls among OPWD

The risk of falls in the older people was measured using the MFS, with a minimum and a maximum of 0 and 100, respectively, with an average score of 23.97 ± 23.54. The basic information on the fall risk score for the elderly is shown in Table 1.

**Table 1:** Basic information on the risk of falls in the older people.

	**Frequency**	**Percentage (%)**
Low risk	488	52.42
Medium risk	287	30.83
High risk	156	16.76
Total	931	100

### Associated factors of fall risk among OPWD

The results of the one-factor analysis (Table 2) show that the differences between the risk groups in terms of the basic health problems, living environment problems, social support issues, and behavioral awareness problems were statistically significant (*p* < 0.05) (Table 2).

**Table 2:** Basic demographic characteristics and health status.

		**Frequency**	**Percentage**	**Fall risk score**	**F**	***P-*** **Value**
**Basic health issues**						
Gender	Males	429	46.08%	22.27 ± 22.35	2.048	0.041
	Female	502	53.92%	25.44 ± 24.45		
Age (in years)	60–70	145	15.57%	18.24 ± 21.01	10.694	<0.001
	70–79	326	35.02%	21.00 ± 22.89		
	80–89	359	38.56%	29.14 ± 24.50		
	90–99	101	10.85%	23.51 ± 22.23		
Types of dementia	Alzheimer's disease	560	72.35%	22.06 ± 23.06	6.668	<0.001
	Vascular dementia	50	6.46%	36.20 ± 27.06		
	Mixed dementia	103	13.31%	27.57 ± 24.31		
	Others	61	7.88%	23.11 ± 20.44		
Number of chronic diseases	No	186	19.98%	15.16 ± 18.60	25.323	<0.001
	A type	249	26.75%	21.39 ± 21.69		
	Various kinds	496	53.28%	28.59 ± 24.95		
Activities of daily living	Life self-care	140	15.09%	5.25 ± 10.98	64.046	<0.001
	Mild dysfunction	279	30.06%	19.01 ± 21.97		
	Moderate dysfunction	143	15.41%	29.30 ± 21.13		
	Severe dysfunction	366	39.44%	32.79 ± 24.04		
**Social support issues**						
Marital status	Spouse present	433	46.51%	23.35 ± 23.95	3.845	0.009
	Divorced	37	3.97%	21.49 ± 24.86		
	Widowed	395	42.43%	26.20 ± 23.51		
	Never married	66	7.09%	16.21 ± 18.08		
Average monthly income	0–1,000	275	29.54%	21.11 ± 22.13	3.231	0.012
	1,000–3,000	206	22.13%	25.22 ± 23.86		
	3,000–5,000	287	30.83%	27.37 ± 23.68		
	5,000–7,000	117	12.57%	21.58 ± 24.10		
	Above 7,000	46	4.94%	20.54 ± 25.65		
Number of children	0	81	8.97%	17.96 ± 19.54	3.291	0.020
	1	202	22.37%	21.51 ± 21.09		
	2	296	32.78%	25.42 ± 24.77		
	Above 3	324	35.88%	26.33 ± 24.51		
Zarit burden interview	<=22.00	25	2.69%	10.20 ± 16.10	11.085	<0.001
	23.00–44.00	239	25.73%	17.64 ± 20.43		
	45.00–66.00	427	45.96%	25.68 ± 24.09		
	67.00–88.00	212	22.82%	27.31 ± 23.58		
	Above 89.00	26	2.80%	38.65 ± 28.72		
The educational level of the caregiver	Illiteracy	52	5.70%	22.31 ± 21.29	4.305	0.005
	Primary school	314	34.43%	23.47 ± 23.58		
	Middle school	433	47.48%	26.77 ± 24.43		
	University and above	113	12.39%	17.88 ± 20.22		
Caregiver knowledge	Not understood	248	27.56%	24.40 ± 24.76	3.869	0.021
	General	347	38.56%	26.56 ± 25.02		
	See	305	33.89%	22.34 ± 20.81		
**Behavioral awareness issues**						
Clinical dementia rating	0	52	5.59%	4.81 ± 12.40	28.652	<0.001
	0.5	193	20.73%	13.91 ± 19.50		
	1	230	24.70%	24.67 ± 23.91		
	2	196	21.05%	28.34 ± 22.56		
	3	260	27.93%	31.38 ± 23.87		
Fall	No	780	83.78%	17.44 ± 17.95	615.994	<0.001
	Yes	151	16.22%	57.75 ± 19.80		
Aspiration	No	824	88.51%	21.24 ± 21.83	108.469	<0.001
	Yes	107	11.49%	45.09 ± 25.61		
Scald	No	876	94.09%	22.99 ± 22.85	26.880	<0.001
	Yes	55	5.91%	39.73 ± 28.57		
Falling out of the bed	No	877	94.20%	22.53 ± 22.53	61.411	<0.001
	Yes	54	5.80%	47.59 ± 27.03		

### Logistic regression analysis of factors related to fall risk among OPWD

Taking the risk level of the falls in old age (1, 2, and 3 = low, medium, and high risk of falls, respectively) as the dependent variable, and the theory of health ecology as the framework, four models were established by adding the factors at various levels; model one, two, and three contained factors at the level of basic health problems, at the level of basic health issues and living environment problems, and the basic health problems, living environment difficulties, and social support problems, respectively; model four encompassed all levels of the factors.

The regression analysis results (Table 3) showed that 80–90 years old, vascular dementia, marital status, and history of falls were risk factors for the elderly's cognitive function; the number of chronic diseases, ADL, living environment, ZBI, Caregiver knowledge, CMAI, and CDR were the protective factors for the risk of falls in the older adults.

**Table 3:** Multiple orderly regression of the risk factors associated with falls (OR).

**Variable**	**Model one**	**Model two**	**Model three**	**Model four**
**Basic health issues**				
Males	0.858	0.959	1.125	1.142
60–69 years old	0.969	0.880	1.099	1.125
70–79 years old	1.394	1.294	1.332	1.215
80–89 years old	1.954^*^	1.840^*^	1.831^*^	2.094^**^
Alzheimer's disease	1.036	0.983	0.839	0.583
Vascular dementia	2.230^*^	2.219^*^	1.904	0.968
Mixed dementia	1.428	1.537	1.480	1.103
No chronic diseases	0.466^***^	0.475^**^	0.440^***^	0.448^**^
A type of chronic diseases	0.685^*^	0.676^*^	0.643^*^	0.680
Activities of daily living = 1.00	0.064^***^	0.074^***^	0.046^***^	0.112^***^
Activities of daily living = 2.00	0.447^***^	0.470^***^	0.400^***^	0.291^***^
Activities of daily living = 3.00	1.051	1.000	0.811	0.587
**Living environment issues**				
Residential environment score		0.963^***^	0.971^*^	0.985
**Social support issues**				
<1,000			0.477	0.660
1,000–3,000			0.678	0.726
3,000–5,000			0.440	0.542
5,000–7,000			0.500	0.494
Spouse present			3.251	4.238^*^
Divorced			5.912^**^	4.641
Widowed			2.474	3.323
Childless			0.845	1.756
A child			0.614	0.743
Two children			0.979	0.870
Zarit burden interview = 1			0.237	1.534
Zarit burden interview = 2			0.266^**^	0.774
Zarit burden interview = 3			0.530	1.186
Zarit burden interview = 4			0.457	0.891
Caregivers are illiterate			0.818	0.859
Primary school education level of the caregivers			1.166	1.551
Caregiver's college education			1.464	1.381
Caregivers are unaware of dementia			1.866^**^	1.603^*^
Caregivers generally know about dementia			1.428	1.502
**Behavioral awareness issues**				
Cohen Mansfield agitation inventory				1.019^***^
Clinical dementia rating = 0				0.000
Clinical dementia rating = 0.5				1.002
Clinical dementia rating = 1.0				1.893^*^
Clinical dementia rating = 2.0				1.784^*^
Fall				0.024^***^
Aspiration				0.667
Scald				1.029
Falling out of the bed				1.553
−2 LL	660.756	1224.272	1203.329	884.644
Cox and nell *R*^2^	0.210	0.232	0.292	0.517
Nagelkerke *R*^2^	0.243	0.267	0.336	0.594

* p < 0.05,

** p < 0.01,

*** p < 0.001.

## Discussion

### Analysis of the current situation of the risk of dementia among OPWD

This study's results indicated that the homebound OPWD in China have a high risk of falling, which is consistent with the previous studies (25). In this research, 16.2% of the older adults had fallen in the last 3 months. According to the MFS, 30.80 and 16.80% of the elderly were at an intermediate and a high risk of falling, respectively. This demonstrated that the current situation of the fall risks among the older people in China is unoptimistic. In the context of the increasing aging, attention should be paid to the intervention regarding the fall risk factors for the older people, improving their quality of life, and reducing the burden of the old-age and medical care in the society.

### Effects of underlying health problems on the risk of falls among OPWD

This study found that age, type of dementia, number of chronic diseases, and the ADL in the basic health problems had a significant impact on the risk of falls in the older people. Moreover, women had a greater risk of falls than men; however, this was not significant in the regression models. In some countries, males were found to be more likely to die from a fall (26), while females suffered more non-fatal falls. Older women were especially prone to falls and an increased injury severity (27). Age has been indicated as a key risk factor for falls (28). The elderly have the highest risk of death or serious injury due to a fall; further this risk increases with age. Older adults aged 80–90 years are at the highest risk of falling. This risk level may be partially due to physical, sensory, and cognitive changes associated with aging, in combination with the environments that are not adapted for an aging population. The reason for the reduced risk in the older people aged over 90 years might be limited daily activities. Kim reported that age was significantly associated with the cognitive frailty-related falls (29).

In this study, vascular dementia was identified as the greatest risk of falls. The cardiovascular and cerebrovascular diseases can cause palpitations, headaches, hemiplegia, and more; if the older people experience sickness while walking, it would easily lead to falls. Ting found that accidental falls in patients with cardiovascular disease usually occur during the winter months and between 12 and 9 a.m. (30). During this period, the incidence of cardiovascular disease is also high.

Multimorbidity was defined as the coexistence of two or more chronic non-communicable diseases in one patient (31). According to this survey data, more than 80% of the elderly have chronic diseases, of which over half of those with diseases have two species and more chronic diseases. Moreover, prior studies have found that the number of people with chronic diseases is a risk factor for falls. The more types of chronic diseases there are, the higher the fall risk score obtained. It could be considered that having more than one chronic disease is a fall risk factor for the older persons. Numerous studies have confirmed that an increased types of chronic diseases may increase the risk of falls (28, 32). It was suggested that strengthening the management of chronic diseases in the elderly community is an important part of home care; furthermore, communities with more older people could often hold the corresponding health management activities to provide nursing services for those with chronic diseases. In the process of home care for the Alzheimer's patients, it is not only necessary to pay attention to the situation of dementia, but also treat and prevent chronic diseases and improve the elderly's physical fitness.

Regarding the patients with dementia, due to memory, learning, thinking, mental, and other obstacles, the condition is often accompanied by the loss of self-care ability in life. They need to have a good balance function in the process of completing daily sitting, standing, transferring, and other actions. The functional limitations, as measured by the ADL scale, might identify considerably higher levels of disability that might explain the protective finding of the multivariate model, contrary to the predictive effect found generally (28); it is marginally protective in the multivariate model. Similarly, Woo identified the relationship between sarcopenia and fall-related injuries in the community-dwelling older adults living in South Korea (33).

### The impact of living environment problems on the risk of falls among OPWD

The living environment is a protective factor against the risk of falls, prompting us to arrange a reasonable home environment for the older people, reduce their safety risks, improve their sense of security, and avoid them from feeling stressed due to the environmental stimulation. Additionally, Neslihan assessed the risk factors for falls in the home environment (34).

### The impact of social support issues on the risk of falls among OPWD

In the establishment of the logistic model, the independent variable of the average monthly income was not significant. This might be because certain economic support could make life convenient; however, under the premise that the pension medical insurance system is more perfect and the basic living needs of older people are guaranteed, the demand for their economic conditions is relatively low.

The elderly with spouses are at a lesser risk of falling than those who are divorced or widowed, however, at a higher risk than those who have never been married. There are some inconsistencies with previous studies (27). Considering the results, the greater the number of children, the higher the risk of falling. Nevertheless, additional research may better explain these anomalous relationships.

Consequently, the caregivers' literacy level has less influence on the fall risk than their actual dementia knowledge level. Yu surveyed the knowledge, attitudes, behaviors, and the related factors of fall prevention among the relatives of the elderly in the community; only 30.4% of the nursing staff scored A in the fall prevention knowledge (35). This indicates that the older people and the nursing staff should actively learn the relevant knowledge and improve the level of care.

### The effect of behavioral awareness problems on the risk of falls among OPWD

The results showed a significant positive correlation between the fall risk and the CMAI. The older adults with dementia exhibited agitated behaviors that could harm patients, others, and their surroundings. The most difficult challenge faced by the caregivers was the OPWD's agitation behaviors, which was observed in nearly 89% of the cases. Improper handling of these behaviors could result in self-injury or harm to others. These agitation behaviors of the OPWD are not only related to the disease factors, but can also be triggered by the caregivers' negative communication styles or by adverse environmental stimuli (36, 37). Furthermore, because the Alzheimer's patients have weak perception and thinking skills, they are susceptible to falling and having other accidents. In this process, we are reminded not only to pay attention to the environmental factors, but also to the psychological state of the older people receiving home care. Those patients with unconsciousness and mental mania can be protected by restraint belts that can effectively avoid them from falling. With the degree of cognitive dysfunction in the patients with the CDR responses, the risk of falls increases with the disease severity, which has also been widely reported (38).

This study's findings suggested that the older adults with a history of falls are at a greater risk of falling. Soyano showed that the fall history may predict future falls (39). This may be because the elderly with a history of falls are more likely to have the fall-related risk factors. If effective precautions are not taken, the likelihood of falling again is greater in such older adults, as compared to those without a history of falls. In addition, whether the patient has experienced aspiration, burns, or falling out of bed also reflects the severity of the fall risk to some extent.

## Data availability statement

The raw data supporting the conclusions of this article will be made available by the authors, without undue reservation.

## Ethics statement

The studies involving human participants were reviewed and approved by the Research Ethics Committee of Ningbo College of Health Sciences (NBWY-2019-012). The patients/participants provided their written informed consent to participate in this study.

## Author contributions

RM and YH planned the study and the overall analysis method. XD and GL substantially contributed to acquisition and interpretation of data and writing of the manuscript. XY contributed to data analysis and data collation. All authors participate in the writing, discussion of the paper, read, and approved the final version of the manuscript.

## Funding

We gratefully acknowledge funding support from the Zhejiang Provincial Natural Science Foundation of China (Grant No. LQ20H260006).

## Conflict of interest

The authors declare that the research was conducted in the absence of any commercial or financial relationships that could be construed as a potential conflict of interest.

## Publisher's note

All claims expressed in this article are solely those of the authors and do not necessarily represent those of their affiliated organizations, or those of the publisher, the editors and the reviewers. Any product that may be evaluated in this article, or claim that may be made by its manufacturer, is not guaranteed or endorsed by the publisher.
