# Supplementary material for: Fall risks and the related factors for the homebound older people with dementia: Evidence from East China
Source: Front Public Health. 2022 Aug 25;10:946097. doi: 10.3389/fpubh.2022.946097 (PMC9458357; doi:10.3389/fpubh.2022.946097)
Supplement: Supplementary file 1 [file Table_2.DOCX]

Appendix 2: Study indicators

| Indicators | type |
| --- | --- |
| Basic health issues | |
| Gender | [categorical variable]  1=male, 2=female |
| Age (in years) | [categorical variable]  1=60-70, 2=70-79, 3=80-89, 4=90-99 |
| Types of dementia | [categorical variable]  1=Alzheimer's disease, 2=Vascular dementia, 3=Mixed dementia, 4=others |
| Number of chronic diseases | [categorical variable]  1=No, 2=A type, 3=Various kinds |
| Activities of daily living | [categorical variable]  1=life self-care, 2=Mild dysfunction, 3=Moderate dysfunction, 4=Severe dysfunction |
| Living environment issues | |
| Residential environment score | [continuous variable] |
| Social support issues | |
| marital status | [categorical variable]  1=spouse present, 2=divorced, 3=widowed, 4=Never married |
| Average monthly income | [categorical variable]  1=0-1000, 2=1000-3000, 3=3000-5000, 4=5000-7000, 5=Above 7000 |
| Number of children | [categorical variable]  1=0, 2=1, 3=2, 4=Above 3 |
| Zarit Burden Interview | [categorical variable]  1=<= 22.00, 2=23.00 - 44.00, 3=45.00 - 66.00, 4=67.00 - 88.00, 5=Above 89.00 |
| The educational level of caregiver | [categorical variable]  1=illiteracy, 2=primary school, 3=middle school, 4=University and above |
| Caregiver knowledge | [categorical variable]  1=not understood, 2=General,3=See |
| Behavioral awareness issues | |
| Clinical Dementia Rating | [categorical variable]  0=0, 0.5=0.5, 1=1, 2=2, 3=3 |
| fall | [categorical variable]  0=No, 1=Yes |
| aspiration | [categorical variable]  0=No, 1=Yes |
| scald | [categorical variable]  0=No, 1=Yes |
| falling out of bed | [categorical variable]  0=No, 1=Yes |
