# Supplementary material for: Fall risks and the related factors for the homebound older people with dementia: Evidence from East China
Source: Front Public Health. 2022 Aug 25;10:946097. doi: 10.3389/fpubh.2022.946097 (PMC9458357; doi:10.3389/fpubh.2022.946097)
Supplement: Supplementary file 2 [file Data_Sheet_1.docx]

Appendix 1: Questionnaires

**Safety Risk Questionnaire of Home Care for Mentally Disabled Elderly**

**Dear old people and caregivers:**

**Hello!**

**This survey is mainly to comprehensively understand the possible security risks of home care for the elderly with dementia, as well as the burden of caregivers, and provide the basis for the government to formulate pension service policies, in order to improve the care service level of the elderly with dementia, and ultimately improve the quality of life and happiness of the elderly. Hope you can truthfully fill in the relevant content, the personal information involved, we will strictly confidential! Thank you for your participation! Have a happy life!**

**Project group**

**A Basic situation** **of the elderly**

**A1 Sex of the elderly [Investigator Observation]:**

1. Male 2. Female

**A2 Age of the elderly: years (based on the past birthday)**

**A3 The educational level of the elderly:**

1. No schooling 2. Primary school 3. Junior high school

4. High school/technical secondary school/vocational school

5. College degree 6. Bachelor degree and above

**A4 Current marital status of the elderly:**

1. Spouse, age 2. Divorce 3. Widows 4. Never married

**A5 Current situation of children of the elderly:**

1. Children 2. No children

**A6 Who does the old man live with now? (Multiple-choice questions)**

1. Living alone 2. Spouses 3. Children 4. Other relatives 5. Nurse 6. Other

**A7 What is the average monthly income of the elderly?**

1. ≤¥1000 2. ¥1001~ ¥3000 3. ¥3001~ ¥5000 4. ¥5001 ~ ¥7000 5. ≥ ¥7001

**A8 What medical insurance does the elderly in enjoy?**

1. No 2. Basic Medical Insurance for Urban Employees

3. Basic Medical Insurance for Urban and Rural Residents (Integration of Basic Medical Insurance for Urban Residents and New Rural Cooperative Medical Scheme)

4. Public Health (Cadre Health, etc.) 5. Commercial Medical Insurance 6. Other

**A9. Is the diagnosis of dementia in elderly clear?**

1.No 2.Yes

**A9.1 Which of the following types is clearly diagnosed?**

1. Alzheimer’ s disease 2. Vascular dementia 3. Mixed dementia

4. Ravel’ s dementia 5. Other types

**A10. Does elderly people take anti - dementia drugs?**

1. not taking 2. one kind a day 3. many kinds a day 4. one kind a week

5. many kinds a week 6. unclear.

**A11. What chronic diseases do the A11 elderly suffer from? (Multiple-choice questions)**

1. No 2.other mental illnesses (depression, schizophrenia, etc.) 3. Hypertension 4. Diabetes 5. Cardiovascular and cerebrovascular diseases 6. Cataract / glaucoma

7. Gastric diseases 8. Osteoarthritis 9. Chronic pulmonary diseases 10. Asthma 11. Malignant tumors 12. Reproductive system diseases 13. Other chronic diseases

**A12 What are the following daily activities of the elderly? [Investigator: Select conformity option in each item]**

| **item** | **1 complete independence** | **2 Partial help** | **3 Great help** | **4 Complete dependences** |
| --- | --- | --- | --- | --- |
| 1. Food intake | 10 | 5 | 0 | ---- |
| 2. Bathing | 5 | 0 | ---- | ---- |
| 3. Modification (washing, brushing, shaving, combing) | 5 | 0 | ---- | ---- |
| 4 Wearing (shoelaces, buttons, etc.) | 10 | 5 | 0 | ---- |
| 5. Control of defecation | 10 | 5 (out of control < 1 time per week) | 0 (out of control) | ---- |
| 6. Control urine | 10 | 5 (out of control < 1 time per week) | 0 (out of control) | ---- |
| 7. Toilet (clean, tidy, flush) | 10 | 5 | 0 | ---- |
| 8. Transfer of beds and chairs | 15 | 10 | 5 | 0 |
| 9. Walking 45 miters flat | 15 | 10 | 5 | 0 |
| 10. Up and down stairs | 10 | 5 | 0 | ---- |

**A13 What is the intelligence status of the elderly? [ Investigator: Select conformity option in each item]**

| item | Dementia-free  CDR0 | Suspicious dementia  CDR0.5 | Mild dementia  CDR1.0 | Moderate dementia  CDR2.0 | Severe dementia  CDR3.0 |
| --- | --- | --- | --- | --- | --- |
| Memory | 1. Forgetfulness without memory impairment or only mildly unstable | 2. Mild and sustained amnesia; partly recalling things | 3. Moderate memory impairment; forgetting about recent events is prominent and impairs the memory of daily activities | 4. Severe memory impairment; remember the familiar things of the past, and the new things quickly forget | 5. Severe memory loss; memory with only fragments |
| Directional force | 1. Completely correct orientation | 2. Completely correct orientation except for minor difficulties in time orientation | 3. Moderate difficulty in timing; directional to inspection sites; it may lose orientation in other locations | 4.Time orientation has serious difficulties; frequent location deorientation | 5. Person orientation only |
| Judgment + problem-solving ability | 1.It can well solve daily problems, handle affairs and finance; good judgment | 2.Slight defect | 3. Moderate difficulties; social judgment is usually preserved | 4.Serious damage; social judgment is usually impaired | 5. Cannot judge or solve problems |
| Social affairs | 1. The same level of independence in work, shopping and community activities as in the past | 2. Minor damage to these activities | 3. Although they also participate, they cannot carry out these activities independently; occasionally normal | 4. Outdoor activities cannot be conducted independently; but it can be taken to outdoor activities | 5. Outdoor activities cannot be conducted independently; the illness cannot be brought to outdoor activities |
| Family + hobbies | 1. Interest in family life, hobbies and intellectual needs is well maintained | 2. Minor damage | 3. Mild obstacles to family activities, abandonment of difficult household tasks, abandonment of complex hobbies and interests | 4. Be able to do simple housework only and maintain very limited scope and level of interest | 5. Loss of meaningful family activities |
| Personal | 1. Fully capable of self-care |  | 3. Need for oversight | 4. Need for assistance in dress, hygiene, personal financial custody | 5.Personal care needs a lot of help; frequent incontinence |

**B. Risk factor assessment**

**[Investigator: Select conformity option in each item]**

| **B1 fall assessment** | **1** | **2** | **3** | **4** | **5** |
| --- | --- | --- | --- | --- | --- |
| **1. Have you fallen in the last three months?** | No | 1–2 times | 3–4 times | 5–6 times | more than 6 times |
| 2. Are there more than one different system medical diagnosis? | No | 2 | 3 | 4 | 4 or more |
| 3. Do you use walking aids? | Not used | Activities supported by people | Using crutches | Using walk aid | Walk by furniture |
| 4. Whether intravenous infusion or heparin lock? | None | Very few | Sometimes | Frequently | Always |
| 5. Is gait normal? | Normal | Bed inactivity | little weak lower limb | Very weak legs | Disability or dysfunction |
| 6. Will you overestimate your ability? | None | Very few | Sometimes | Frequently | Always |

**B3 Has any aspiration occurred in the past three months?**

1. None 2. 1-2 times 3. 3-4 times 4. 5-6 times 5. More than 6 times

**B4 Has the elderly been scalded in recent three months?**

1. None 2. 1-2 times 3. 3-4 times 4. 5-6 times 5. More than 6 times

**B5 Has the elderly fallen into bed in the past three months?**

1. None 2. 1-2 times 3. 3-4 times 4. 5-6 times 5. More than 6 times

**B6 Passion Behavior Assessment (CMAI)** [Note: A review of the past two weeks of observed acts of agitation, according to the frequency of occurrence ' never appeared a specific act of agitation ' to ' several times per hour the act of agitation ' in turn 1 to 7 points.]

| **item** | None | 1 in nearly 2 weeks | 1 per week | Multiple weekly | Almost every day | Multiple times a day | Multiple per hour |
| --- | --- | --- | --- | --- | --- | --- | --- |
| 1.Walking or wandering purposeless | 1 | 2 | 3 | 4 | 5 | 6 | 7 |
| 2.Inappropriate dress or coat | 1 | 2 | 3 | 4 | 5 | 6 | 7 |
| 3.Spit at will (including meals) | 1 | 2 | 3 | 4 | 5 | 6 | 7 |
| 4.curse others or intimidate or insult others in words | 1 | 2 | 3 | 4 | 5 | 6 | 7 |
| 5.Irrational requests for attention or assistance | 1 | 2 | 3 | 4 | 5 | 6 | 7 |
| 6.Repeated speaking or questioning | 1 | 2 | 3 | 4 | 5 | 6 | 7 |
| 7.Strike or self | 1 | 2 | 3 | 4 | 5 | 6 | 7 |
| 8.Kicking or objects | 1 | 2 | 3 | 4 | 5 | 6 | 7 |
| 9.Catch others or themselves or objects. | 1 | 2 | 3 | 4 | 5 | 6 | 7 |
| 10.Push others away | 1 | 2 | 3 | 4 | 5 | 6 | 7 |
| 11.Tossing items (including food) or sweeping them off the table | 1 | 2 | 3 | 4 | 5 | 6 | 7 |
| 12.An unusual sound (strange laughter or crying) | 1 | 2 | 3 | 4 | 5 | 6 | 7 |
| 13.Scream, shout or howl | 1 | 2 | 3 | 4 | 5 | 6 | 7 |
| 14.Bite or object | 1 | 2 | 3 | 4 | 5 | 6 | 7 |
| 15.To be close to or hold on to others | 1 | 2 | 3 | 4 | 5 | 6 | 7 |
| 16.Unprovoked departure or unauthorized access to other places | 1 | 2 | 3 | 4 | 5 | 6 | 7 |
| 17.deliberate fall | 1 | 2 | 3 | 4 | 5 | 6 | 7 |
| 18.Complaints or complaints | 1 | 2 | 3 | 4 | 5 | 6 | 7 |
| 19.Negative | 1 | 2 | 3 | 4 | 5 | 6 | 7 |
| 20.Eating and drinking non-food items | 1 | 2 | 3 | 4 | 5 | 6 | 7 |
| 21.Injuring oneself or others | 1 | 2 | 3 | 4 | 5 | 6 | 7 |
| 22.Unproper handling of things (random search of drawers, unauthorized access to other people 's objects or untouchables) | 1 | 2 | 3 | 4 | 5 | 6 | 7 |
| 23.Hidden objects | 1 | 2 | 3 | 4 | 5 | 6 | 7 |
| 24.Storage or collection of excessive or improper items | 1 | 2 | 3 | 4 | 5 | 6 | 7 |
| 25.Torn or destroyed objects / property | 1 | 2 | 3 | 4 | 5 | 6 | 7 |
| 26.Repeated actions (shaking body, rubbing body or object, tapping objects, and ripping skin) | 1 | 2 | 3 | 4 | 5 | 6 | 7 |
| 27.Oral requests | 1 | 2 | 3 | 4 | 5 | 6 | 7 |
| 28.Sexual behaviors | 1 | 2 | 3 | 4 | 5 | 6 | 7 |
| 29.Restless or restless | 1 | 2 | 3 | 4 | 5 | 6 | 7 |

**C Assessment of the surrounding living environment**

|  |  |  |  |  |  |
| --- | --- | --- | --- | --- | --- |
| 1.Can the home environment reduce the security risks of the elderly and improve their sense of security? | Not at all | Not very competent | General | Almost | Completely able |
| 2.Can the home environment strengthen the elderly 's clear identification of living space, time and sociality? | Not at all | Not very competent | General | Almost | Completely able |
| 3.Can home environment and care strategies support older persons in improving their life skills, including movement, washing, bathing, such as toilets, diet, etc.? | Not at all | Not very competent | General | Almost | Completely able |
| 4.Can the home environment support the elderly in social activities in public spaces? | Not at all | Not very competent | General | Almost | Completely able |
| 5.Can home environment provide private space such as rest for the elderly? | Not at all | Not very competent | General | Almost | Completely able |
| 6.Can the home environment support older persons to exercise their personal preferences, choices and decide when to do something? | Not at all | Not very competent | General | Almost | Completely able |
| 7.Whether light, color, sound and other environmental stimuli are adapted to the elderly to avoid the pressure of the elderly due to environmental stimuli? | Not at all | Not very competent | General | Almost | Completely able |
| 8.Whether the current living environment can be associated with the old people's previous living environment, and feel the past and present their own contact? | Not at all | Not very competent | General | Almost | Completely able |

**D Basic situation of main caregivers**

**D1 Your relationship with the elderly:**

1. Spouse 2. Son 3. Daughter-in-law 4. Daughter 5. Son-in-law

6. Brothers and sisters 7. Others

**D2 Your Gender [Investigator observation and filling]:**

1. Men 2. Women

**D3 What is your age? Years old (based on the past birthday)**

**D4 Your educational level:**

1. No schooling 2. Primary school 3. Junior high school

4. High school/technical secondary school/vocational school 5. College degree

6. Bachelor degree and above

**D5 Do you understand the knowledge and skills of dementia care:**

1. I don't know at all 2. I don't know very well 3. Generally 4. I know better 5. I know very well

**D6 burden of care for the elderly: (Zarit Nursing Burden Scale)**

| **In the following questions, please √ on the most appropriate code you think** | **Never** | **Very few** | **Sometimes** | **Frequently** | **Always** |
| --- | --- | --- | --- | --- | --- |
| 1.Do you think that the patients you care for will ask for too much care? | 1 | 2 | 3 | 4 | 5 |
| 2.Do you think that nursing patients will make their time insufficient? | 1 | 2 | 3 | 4 | 5 |
| 3.Do you think there is pressure between taking care of patients and working hard at home? | 1 | 2 | 3 | 4 | 5 |
| 4.Do you think it is difficult because of the patient’s behavior? | 1 | 2 | 3 | 4 | 5 |
| 5.Do you think that patients are troubled with you? | 1 | 2 | 3 | 4 | 5 |
| 6.Do you think that your patient has affected your relationship with your family and friends? | 1 | 2 | 3 | 4 | 5 |
| 7.Do you worry about the future of patients? | 1 | 2 | 3 | 4 | 5 |
| 8.Do you think that patients depend on you? | 1 | 2 | 3 | 4 | 5 |
| 9.Do you feel nervous when the patient is around you? | 1 | 2 | 3 | 4 | 5 |
| 10.Do you think that your health is affected by nursing patients? | 1 | 2 | 3 | 4 | 5 |
| 11.Do you think that because of nursing patients, you have no time to do your own private affairs? | 1 | 2 | 3 | 4 | 5 |
| 12.Do you think your social interaction is affected by caring for patients? | 1 | 2 | 3 | 4 | 5 |
| 13.Do you give up the idea of inviting friends to come home because the patient is at home? | 1 | 2 | 3 | 4 | 5 |
| 14.Do you think that the patient only expects your care, and you seem to be the only person he / she can rely on? | 1 | 2 | 3 | 4 | 5 |
| 15.Do you think you have no money to care for patients except for your expenses? | 1 | 2 | 3 | 4 | 5 |
| 16.Do you think that you may spend more time nursing patients? | 1 | 2 | 3 | 4 | 5 |
| 17.Do you think it is impossible to live in accordance with your will since the beginning of care? | 1 | 2 | 3 | 4 | 5 |
| 18.Do you wish to leave the patient to others for care? | 1 | 2 | 3 | 4 | 5 |
| 19.Do you have any idea what is good for patients? | 1 | 2 | 3 | 4 | 5 |
| 20.Do you think more should be done for patients? | 1 | 2 | 3 | 4 | 5 |
| 21.Do you think you can do better in caring for patients? | 1 | 2 | 3 | 4 | 5 |
| 22.Overall, how do you evaluate your burden on care? | None | light | Middle | heavy | Extremely heavy |

Questionnaires in Chinese

**失智老人居家照护安全风险调查问卷**

**尊敬的老人家和照护者：**

**您好！**

**本次调查主要是为了全面了解失智老人居家照护可能存在的安全风险，以及照护者的负担，为政府制定养老服务政策提供依据，以提高失智老人的照护服务水平, 最终提升老人的生活质量和幸福感。希望您能如实填写相关内容，所涉及的个人信息，我们将严格保密！对于您的参与，我们深表感谢！祝您生活愉快！**

**项目组**

**A 老人基本情况**

**A1老人的性别**【调查员观察填写】：①男 ②女

**A2老人的年龄**：__ __周岁（以刚过去的生日为准）

**A3 老人的文化程度：**

1. 未上过学 2.小学 3.初中 4.高中/中专/职高 5.大学专科 6.本科及以上

**A4 老人现在的婚姻状况：**

1. 有配偶，配偶年龄_______周岁？ 2. 离婚 3. 丧偶 4. 从未结婚

**A5老人现在的子女情况：** 1.有子女_____ 人 2. 无子女

**A6现在老人和谁住在一起（同吃同住）？（多选题）**

1.单独居住 2.配偶 3.子女 4.其他亲属 5.保姆 6.其他

**A7平均每月，老人个人总收入是多少（元）？**

1. ≤1000元 2.1001元~3000元 3.3001元~5000元 4.5001元~7000元 5. ≥7001元

**A8老人享受了什么医疗保险？**

1.无 2. 城镇职工基本医疗保险 3.城乡居民基本医疗保险（城镇居民基本医疗保险与新农合合一） 4. 公费医疗（干部保健等） 5. 商业医疗保险 6. 其他

**A9老人的失智症是否明确诊断？** 1.否 2.是，确诊时间： 年。

A9.1明确诊断为下列哪一类型？

1.阿尔茨海默病 2.血管性失智症 3.[混合型失智症](javascript:void(0)) 4.雷维氏体失智症 5.其他型

**A10老人是否服用抗失智症药物？**

1.不服用 2.每天1种 3.每天多种 4.每周1种 5.每周服用多种 6.不清楚

**A11老人同时患有哪些慢性病？（多选题）**

1.无 2. 其他精神方面疾病（抑郁、精神分裂等） 3.高血压 4.糖尿病

5.心脑血管疾病 6.白内障/ 青光眼 7.胃病 8.骨关节病 9.慢性肺部疾病 10.哮喘 11.恶性肿瘤 12.生殖系统疾病 13.其他慢性病

**A12老人进行下列日常活动时属于哪种情况？【调查员：在每一项中选符合选项】**

| **项 目** | **①完全独立** | **②需部分帮助** | **③需极大帮助** | **④完全依赖** |
| --- | --- | --- | --- | --- |
| **1.进食** | 10 | 5 | 0 | ---- |
| **2.洗澡** | 5 | 0 | ---- | ---- |
| **3.修饰（洗脸、刷牙、刮脸、梳头）** | 5 | 0 | ---- | ---- |
| **4.穿衣（系鞋带、纽扣等）** | 10 | 5 | 0 | ---- |
| **5.控制大便** | 10 | 5（每周<1次失控） | 0（失控） | ---- |
| **6.控制小便** | 10 | 5（每周<1次失控） | 0（失控） | ---- |
| **7.如厕（擦净、整理衣裤、冲水）** | 10 | 5 | 0 | ---- |
| **8.床椅转移** | 15 | 10 | 5 | 0 |
| **9.平地行走45米** | 15 | 10 | 5 | 0 |
| **10.上下楼梯** | 10 | 5 | 0 | **----** |

**A13老人的智力状况属于下列哪种情况？【调查员：在每一项中选符合选项】**

| **项目** | **无痴呆CDR0** | **可疑痴呆CDR0.5** | **轻度痴呆CDR1.0** | **中度痴呆CDR2.0** | **重度痴呆CDR3.0** |
| --- | --- | --- | --- | --- | --- |
| **记忆力** | 1.无记忆力缺损或只有轻度不恒定的健忘 | 2.轻度、持续的健忘；对事情能部分回忆 | 3.中度记忆缺损；对近事遗忘突出，有碍日常活动的记忆缺损 | 4.严重记忆缺损；能记住过去非常熟悉的事情，新事务则很快遗忘 | 5.严重记忆丧失；仅存片断的记忆 |
| **定向力** | 1.能完全正确定向 | 2.除时间定向有轻微困难外，其他能完全正确定向 | 3.时间定向有中度困难；对检查场所能定向；在其他地点可能失定向 | 4.时间定向有严重困难；常有地点失定向 | 5.仅有人物定向 |
| **判断力+解决问题能力** | 1.能很好解决日常问题、处理事务和财务；判断力良好 | 2.有轻微缺损 | 3.有中度困难；社会判断力通常保存 | 4.有严重损害；社会判断力通常受损 | 5.不能做出判断，或不能解决问题 |
| **社会事务** | 1.在工作、购物、社团活动等方面独立水平与过去相同 | 2.在这些活动方面有轻微损害 | 3.虽然还参加但已不能独立进行这些活动；偶尔检查表现正常 | 4.不能独立进行室外活动；但可被带到室外活动 | 5.不能独立进行室外活动；病重得不能被带到室外活动 |
| **家庭+爱好** | 1.家庭生活、爱好和需用智力的兴趣均很好保持 | 2.轻微受损 | 3.家庭活动轻度障碍，放弃难度大的家务，放弃复杂的爱好和兴趣 | 4.仅能作简单家务，兴趣保持范围和水平非常有限 | 5.丧失有意义的家庭活动 |
| **个人料理** | 1.完全有能力自我照料 |  | 3.需要督促 | 4.在穿着、卫生、个人财务保管方面**需要帮助** | 5.个人料理**需要很多帮助**；经常二便失禁 |

**B、风险因素评估**

**【调查员：在每一项中选符合选项】**

| **B1跌倒情况评估** |  |  |  |  |  |
| --- | --- | --- | --- | --- | --- |
| **1.近三个月是否跌倒过？** | 无 | 有1-2次 | 有3-4次 | 有5-6次 | 有6次以上 |
| 2.是否有超过1个的不同系统医学诊断？ | 无 | 有2个 | 有3个 | 有4个 | 有4个以上 |
| 3.是否使用行走辅助用具？ | 不使用 | 活动由人搀扶 | 使用拐杖 | 使用助行器 | 扶靠家具行走 |
| 4.是否静脉输液或使用肝素锁？ | 无 | 很少 | 有时 | 经常 | 总是 |
| 5.步态是否正常？ | 正常 | 卧床不能活动 | 双下肢有点软弱乏力 | 双下肢非常软弱乏力 | 残疾或功能障碍 |
| 6.是否会高估自己的能力？ | 无 | 很少 | 有时 | 经常 | 总是 |

**B3近三个月是否发生过误吸？**1.无 2.有1-2次 3.有3-4次 4.有5-6次 5.有6次以上

**B4近三个月老人是否烫伤过?** 1.无 2.有1-2次 3.有3-4次 4.有5-6次 5.有6次以上

**B5近三个月老人是否坠床过?** 1.无 2.有1-2次 3.有3-4次 4.有5-6次 5.有6次以上

**B6激越行为评估（CMAI量表）【说明：对前2周观察到的激越行为进行回顾，按照发生的频次以“从来没有出现特定的激越行为”到“每个小时出现几次该激越行为”依次计 1～7分。】**

| **项 目** | 无 | 近2周  1次 | 每周1次 | 每周多次 | 几乎每天出现 | 每天多次 | 每小时多次 |
| --- | --- | --- | --- | --- | --- | --- | --- |
| 1.踱步或无目的的徘徊 | 1 | 2 | 3 | 4 | 5 | 6 | 7 |
| 2.不恰当的穿衣或宽衣 | 1 | 2 | 3 | 4 | 5 | 6 | 7 |
| 3.随意吐痰（包括在进餐中） | 1 | 2 | 3 | 4 | 5 | 6 | 7 |
| 4.咒骂别人或在言语上恐吓或侮辱别人 | 1 | 2 | 3 | 4 | 5 | 6 | 7 |
| 5.为求注意或帮助而作出无理要求 | 1 | 2 | 3 | 4 | 5 | 6 | 7 |
| 6.重复说话或提问 | 1 | 2 | 3 | 4 | 5 | 6 | 7 |
| 7.打人或自己 | 1 | 2 | 3 | 4 | 5 | 6 | 7 |
| 8.踢人或物件 | 1 | 2 | 3 | 4 | 5 | 6 | 7 |
| 9.抓别人或自己或物件 | 1 | 2 | 3 | 4 | 5 | 6 | 7 |
| 10.推开别人 | 1 | 2 | 3 | 4 | 5 | 6 | 7 |
| 11.乱掷物品（包括食物）或从桌面上扫落物品 | 1 | 2 | 3 | 4 | 5 | 6 | 7 |
| 12.发出异常声音（奇怪的笑声或哭泣） | 1 | 2 | 3 | 4 | 5 | 6 | 7 |
| 13.尖叫、叫喊或哀嚎 | 1 | 2 | 3 | 4 | 5 | 6 | 7 |
| 14.咬人或物件 | 1 | 2 | 3 | 4 | 5 | 6 | 7 |
| 15.紧靠或抓紧别人 | 1 | 2 | 3 | 4 | 5 | 6 | 7 |
| 16.无故离去或擅自进入其他地方 | 1 | 2 | 3 | 4 | 5 | 6 | 7 |
| 17.蓄意跌倒 | 1 | 2 | 3 | 4 | 5 | 6 | 7 |
| 18.投诉或抱怨 | 1 | 2 | 3 | 4 | 5 | 6 | 7 |
| 19.消极 | 1 | 2 | 3 | 4 | 5 | 6 | 7 |
| 20.吃喝非食品类的东西 | 1 | 2 | 3 | 4 | 5 | 6 | 7 |
| 21.弄伤自己或别人 | 1 | 2 | 3 | 4 | 5 | 6 | 7 |
| 22.不适当地处理东西（乱搜抽屉、擅取别人的物件或摸不该摸的东西） | 1 | 2 | 3 | 4 | 5 | 6 | 7 |
| 23.藏匿物件 | 1 | 2 | 3 | 4 | 5 | 6 | 7 |
| 24.储存或收集过多或不当的物品 | 1 | 2 | 3 | 4 | 5 | 6 | 7 |
| 25.撕破或破坏物件 / 财物 | 1 | 2 | 3 | 4 | 5 | 6 | 7 |
| 26.重复动作（摇动身子、磨擦身体或对象、轻敲物件、轻扯皮肤） | 1 | 2 | 3 | 4 | 5 | 6 | 7 |
| 27.提出口头性要求 | 1 | 2 | 3 | 4 | 5 | 6 | 7 |
| 28.行为性欲表现 | 1 | 2 | 3 | 4 | 5 | 6 | 7 |
| 29.烦躁或坐立不安 | 1 | 2 | 3 | 4 | 5 | 6 | 7 |

**C居住环境评估**

|  |  |  |  |  |  |
| --- | --- | --- | --- | --- | --- |
| 1.居家环境是否能够降低老人安全风险，**提高安全感**？ | 完全不能 | 不太能 | 一般 | 差不多 | 完全能够 |
| 2.居家环境是否能够加强老人对住所空间、时间、社会性的**清晰辨识**？ | 完全不能 | 不太能 | 一般 | 差不多 | 完全能够 |
| 3.居家环境和照护策略是否能够支持老人**提高生活技能，**包括行动、洗漱、洗澡、如厕、饮食等？ | 完全不能 | 不太能 | 一般 | 差不多 | 完全能够 |
| 4.居家环境是否能够支持老人在**公共空间开展社交活动**？ | 完全不能 | 不太能 | 一般 | 差不多 | 完全能够 |
| 5.居家环境是否能够为老人提供休息等**私密空间**？ | 完全不能 | 不太能 | 一般 | 差不多 | 完全能够 |
| 6.居家环境是否能够支持老人**行使个人的偏好、选择**，决定何时做什么事？ | 完全不能 | 不太能 | 一般 | 差不多 | 完全能够 |
| 7.光线、颜色、声音等**环境刺激水平**是否与老人相适应，避免老人因环境刺激而感到压力？ | 完全不能 | 不太能 | 一般 | 差不多 | 完全能够 |
| 8.现居住环境是否能够与与老人以往居住**环境相联系，**感受过去的自己和现在的自己的联系？ | 完全不能 | 不太能 | 一般 | 差不多 | 完全能够 |

**D主要照护者基本情况**

**D1 您与老人的关系：**

1. 配偶 2. 儿子 3. 儿媳 4. 女儿 5. 女婿 6. 兄弟姐妹 7. 其他

**D2 您的性别【调查员观察填写】：** 1.男 2.女

**D3 您的年龄是多少？**____________周岁（以刚过去的生日为准）

**D4 您的文化程度**：

1. 未上过学 2.小学 3.初中 4.高中/中专/职高 5.大学专科 6.本科及以上

**D5您是否了解失智症照护相关常识和技能：**

1.一点不了解 2.不太了解 3.一般 4.较了解 5.非常了解

**D6 照护老人的负担情况：（Zarit 护理负担量表）**

| **请在以下各问题中在您认为最合适代码上打√** | **从不** | **很少** | **有时** | **经常** | **总是** |
| --- | --- | --- | --- | --- | --- |
| 1.您是否认为, 您所照料的病人会向您提出过多的照顾要求? | 1 | 2 | 3 | 4 | 5 |
| 2.您是否认为, 由于护理病人会使自己的时间不够? | 1 | 2 | 3 | 4 | 5 |
| 3.您是否认为, 在照料病人和努力做好家务及工作之间, 你会感到有压力? | 1 | 2 | 3 | 4 | 5 |
| 4.您是否认为, 因病人的行为而感到为难? | 1 | 2 | 3 | 4 | 5 |
| 5.您是否认为, 有病人在您身边而感到烦恼? | 1 | 2 | 3 | 4 | 5 |
| 6.您是否认为, 您的病人已经影响到了您和您的家人与朋友间的关系? | 1 | 2 | 3 | 4 | 5 |
| 7.您对病人的将来, 感到担心吗? | 1 | 2 | 3 | 4 | 5 |
| 8.您是否认为, 病人依赖于您? | 1 | 2 | 3 | 4 | 5 |
| 9.当病人在您身边时, 您感到紧张吗? | 1 | 2 | 3 | 4 | 5 |
| 10.您是否认为, 由于护理病人, 您的健康受到影响? | 1 | 2 | 3 | 4 | 5 |
| 11.您是否认为, 由于护理病人, 您没有时间办自己的私事? | 1 | 2 | 3 | 4 | 5 |
| 12.您是否认为, 由于护理病人, 您的社交受到影响? | 1 | 2 | 3 | 4 | 5 |
| 13.您有没有由于病人在家, 放弃请朋友来家的想法? | 1 | 2 | 3 | 4 | 5 |
| 14.您是否认为, 病人只期盼着您的照料, 您好象是他/她唯一可依赖的人? | 1 | 2 | 3 | 4 | 5 |
| 15.您是否认为, 除外您的花费, 您没有余钱用于护理病人? | 1 | 2 | 3 | 4 | 5 |
| 16.您是否认为, 您有可能花更多的时间护理病人? | 1 | 2 | 3 | 4 | 5 |
| 17.您是否认为, 开始护理以来, 按照自己的意愿生活已经不可能了? | 1 | 2 | 3 | 4 | 5 |
| 18.您是否希望, 能把病人留给别人来照料? | 1 | 2 | 3 | 4 | 5 |
| 19.您对病人有不知如何是好的情形吗? | 1 | 2 | 3 | 4 | 5 |
| 20.您认为应该为病人做更多的事情是吗? | 1 | 2 | 3 | 4 | 5 |
| 21.您认为在护理患者上您能做的更好吗? | 1 | 2 | 3 | 4 | 5 |
| 22.综合看来您怎样评价自己在护理上的负担? | 无 | 轻 | 中 | 重 | 极重 |
